# Lymph node imaging using novel simultaneous PET/MRI and dual-modality imaging agent

**DOI:** 10.1186/2197-7364-2-S1-A53

**Published:** 2015-05-18

**Authors:** Guen Bae Ko, Jae Sung Lee, Hyun Suk Yoon, Daehong Kim, Kyeong Yun Kim, Min Sun Lee, Bo Yeun Yang, Jae Min Jeong, Dong Soo Lee, In Chan Song, Seok-ki Kim

**Affiliations:** Seoul National University, Korea; National Cancer Center, Korea

Lymph node (LN) imaging has clinical significance because the invasion status of the LN is crucial information for disease stratification, staging, and management. Compared with conventional method, simultaneous PET/MRI using the multi-modal imaging marker can offer synergistic advantages. Here, we present LN mapping using selfdeveloped PET/MRI and novel bimodal biomarker. The SiPM-based PET insert which has peak sensitivity of 3.4% and center volumetric resolution of 0.57 cubic mm was developed. The PET insert was placed between the RF and gradient coil of Bruker 7T MRI. For LN targeting, 64Cu-NOTA-ironoxide-mannose, a new LN targeted dualmodality probe yielding superior T2 contrast was used. The relaxivity of the probe was measured by phantom study. Before the simultaneous imaging, MRI of the left and right popliteal lymph node of an anaesthetized BALB/c mouse was acquired as the control. 10 μL of the tracer was then injected into the left hindpaw of same mouse. Simultaneous PET/MRI was acquired for 10 minutes after 10-min and 120-min uptake period. The r2 of the probe was 845.3 at 7T magnetic field. The simultaneous PET/MRI has sufficient resolution and sensitivity to imaging tiny organ like mouse LN. A left popliteal LN in the 120-min post-injection MRI resulted in a remarkable signal decrease compared to those in the pre-injection MRI and that was good agreement with PET. The 10-min post-injection PET also showed clear regional activity in the LN, but the 10-min uptake period was not sufficient to generate MR contrast. It is due to the large difference in the sensitivities of the two modality. The simultaneous PET/MRI is useful for in vivo imaging and bimodal imaging probe development particularly to generating negative T2 contrast.

